# BK ZERO isoform HEK293 stably transfected cell lines differing 3’UTRs to assess miR-9 regulation

**DOI:** 10.1371/journal.pone.0298966

**Published:** 2024-03-19

**Authors:** Katherine Cordero Padilla, Gerardo Alvarado Monefeldt, Adriel Guevárez Galán, Hector G. Marrero, Mario E. Lloret-Torres, Cristina Velázquez-Marrero

**Affiliations:** 1 Department of Anatomy and Neurobiology, University of Puerto Rico Medical Sciences Campus, San Juan, Puerto Rico; 2 Institute of Neurobiology, University of Puerto Rico Medical Sciences Campus, San Juan, Puerto Rico; 3 Windsor University School of Medicine, St. Kitts, West Indies; 4 Department of Biology, University of Puerto Rico Cayey Campus, Cayey, Puerto Rico; 5 Samuel Merritt University, Oakland, California, United States of America; 6 Department of Biology, University of Puerto Rico Bayamón Campus, Bayamón, Puerto Rico; Penn State Health Milton S Hershey Medical Center, UNITED STATES

## Abstract

Research has identified the large conductance voltage- and calcium-activated potassium channel (BK) as a key regulator of neuronal excitability genetically associated to behavioral alcohol tolerance. Sensitivity to ethanol at the molecular level is characterized by acute potentiation of channel activity. BK isoforms show variations in alcohol sensitivity and are differentially distributed on the plasma membrane surface in response to prolonged exposure. MicroRNA (MiRNA) targeting of alcohol-sensitive isoforms coupled with active internalization of BK channels in response to ethanol are believed to be key in establishing homeostatic adaptations that produce persistent changes within the plasma membrane of neurons. In fact, microRNA 9 (miR-9) upregulated expression is a key event in persistent alcohol tolerance mediating acute EtOH desensitization of BK channels. The exact nature of these interactions remains a current topic of discussion. To further study the effects of miR-9 on the expression and distribution of BK channel isoforms we designed an experimental model by transfecting human BK channel isoforms ZERO heterologous constructs in human embryonic kidney cells 293 (HEK293) cells respectively expressing 2.1 (miR-9 responsive), 2.2 (unresponsive) and control (no sequence) 3’untranslated region (3’UTR) miRNA recognition sites. We used imaging techniques to characterize the stably transfected monoclonal cell lines, and electrophysiology to validate channel activity. Finally, we used immunocytochemistry to validate isoform responsiveness to miR-9. Our findings suggest the cell lines were successfully transfected to express either the 2.1 or 2.2 version of ZERO. Patch clamp recordings confirm that these channels retain their functionality and immunohistochemistry shows differential responses to miR-9, making these cells viable for use in future alcohol dependence studies.

## Introduction

Alcohol use disorder (AUD) is defined as an impaired ability to stop or control alcohol use despite adverse social, occupational, or health consequences [1]. AUD is responsible for approximately 140,000 annual deaths in the United States making it the fourth leading cause of preventable deaths in the country [2]. Repeated consumption of alcohol leads to the development of tolerance, referring to the process by which a person adapts to the continued presence of the drug, diminishing the response to it, and becoming dependent on having the substance to be able to function properly [3]. Understanding tolerance at a molecular level may provide insight into addiction and lead to the development of new treatment alternatives for AUD.

One potential mechanism for alcohol tolerance involves the large potassium (BK) channel. The BK channel is a large-conductance calcium and voltage-activated potassium channel that is expressed in the central nervous system (CNS), which is encoded by a single *Slo1* gene (also called *KCa1*.*1 or KCNMA1)* [4]. Single nucleotide polymorphisms of the *Slo1* gene in humans are associated with heavy alcohol drinking [5]. BK is known for its potentiation (20-25mM) after alcohol exposure [6–13]. These effects appear to be region-specific as BK reduces neuronal firing in Globus Palidus Externus neurons [14]. At the behavioral level, inhibition of BK leads to locomotor impairment during ethanol withdrawal in *C*. *elegans* [15]. Changes in locomotion have also been associated with a reduction in clustered BK [16]. Additionally, BK can be activated by other alcohol metabolites such as acetic acid which increases its open probabilities (NP_o_) [17]. Another important interaction between ethanol and BK is isoform-dependent redistribution in the cell membrane [18]. Alternative splicing of *slo-1* is known to greatly alter function [19]. Specifically, BK α insertless (ZERO) isoforms are internalized after 6 hours of alcohol exposure, whereas α isoforms containing the stress-regulated exon (STREX) (a 58 amino acid sequence containing a C-terminal insert that speeds activation, slows down deactivation, and increases open probabilities at negative-voltage potentials) [20, 21] are insensitive to alcohol and thus become predominantly expressed after 6 hours of ethanol exposure [22, 23].

Redistribution of BK after ethanol exposure appears to be reliant on interactions with regulatory microRNA-9 (miR-9) [18, 24]. MiR-9 may interact with BK channels containing a microRNA recognition element (MRE) binding site in their 3^’^UTR (2.1) as a post-transcriptional regulation/modification. This is consistent with studies on STREX channels which have shown the presence of multiple 3’UTRs in neurons with only some of them containing the miR-9 MRE [18]. While miR-9’s role in certain disease states such as cancer is well documented [25]. Its exact role in the development of alcohol tolerance through BK channel internalization remains unclear, though it appears to involve exosomal transport in microglia [26]. Effects at the protein level are also understudied with multiple proteins such as Beta-catenin and ERG-28 suggested as potentially playing a role in BK expression [27, 28] and internalization [29]. To study the effects and mechanisms of miR-9 regulation on BK surface expression degradation and function after alcohol exposure we designed a heterologous expression model using human embryonic kidney (HEK293) cells transfected to express either ZERO channels with 2.1 3^’^UTR’s (responsive to miR-9), ZERO channels with 2.2 3^’^UTR’s (unresponsive to miR-9), or ZERO channels with no 3^’^UTR’s (Restriction Enzyme Site (RES) or Control). The signaling mechanisms mediating alcohol molecular tolerance have been previously characterized in HEK293 and corroborated in isolated mouse neuronal cultures from the hippocampus and striatum [22, 30, 31]. This has allowed for careful characterization in a robust and fast-growing immortalized human cell background which facilitated high levels of recombinant protein using plasmid vectors. We then corroborated channel transfection with confocal microscopy, channel function using patch clamp electrophysiology, and responsiveness to miR-9 using immunocytochemistry.

## Materials & methods

### Cell culture protocols

Human Embryonic Kidney Cells (HEK293) incubation was performed as per manufacturer recommendations (https://www.thermofisher.com/pr/en/home/references/gibco-cell-culture-basics/cell-culture-protocols/cell-culture-useful-numbers.html). HEK293 cells were obtained from American Type Culture Collection (ATCC, CRL-1573, VA, USA). Cells were plated on 35mm petri dishes (Corning 353001, NY, USA) and allowed to grow to 70–90% confluency. Cells were grown in Dulbecco’s modified Eagle’s medium (DMEM) (D5796, Milwaukee, WI, USA) supplemented with 10% fetal bovine serum (FBS) (F-4135), 10 units and 10μg/mL of Penicillin/Streptomycin (Pen/Strep—P4333), 1mM Na^+^-pyruvate (8636) and 2.5 mM HEPES (H0887), (Sigma-Aldrich, INC., St. Louis, MO, USA), at 37°C in humidified 5% CO_2_ incubator. Cell culture media was replaced every 3–5 days.

Methods for the subculturing of stably transfected HEK293 cells were as described in Velazquez-Marrero et al., 2016 [29]. Briefly, cells were plated at a density of 0.3 x 10^6^ cells seeding density in 35mm petri dishes (Corning, NY, USA) and were not allowed to exceed 1.2 x 10^6^ cells in each 35mm petri dish (surface area of 8.8cm^2^ per plate). Once cells reached the desired confluency (70–90%), subculture procedure started by adding 1:10 Tripsin-EDTA (Thermofisher, 15400–054 New York, NY, USA) diluted with 1X Phosphate Buffered Saline (PBS) (Sigma Aldrich, 806552 Milwaukee, WI, USA). DMEM-supplemented media was used to prevent further cell lysis, and centrifuged at 1,380g for 60secs. The supernatant was aspirated and resuspended carefully with 1mL DMEM-supplemented media. Cell counts were then performed using a small 10μL sample of resuspended cells mixed 1:1 with Trypan Blue dye (BioRad, 64315131, UK). Samples were counted with an automated cell counter (BioRad TC20 Singapore) and plated at 0.3 x 10^6^ cell seeding density onto 35mm petri dishes (Corning, NY, USA). Cells are incubated in a Sanyo CO_2_ incubator (MCO-18AC (UV), Sanyo Electric, Japan) at 37.0°C and 5.0% CO_2_ level.

### Transfections

HEK293 cells were transfected at a 70–90% confluence, starting with 3 washes with 1X PBS, and placed in serum-deprived DMEM (without FBS and Pen/Strep) for 24hrs. Transfections were performed using OptiMEM (A41248-02) (Thermofisher, NY, USA) and Lipofectamine 2000 (11668027), (Thermofisher, NY, USA). Briefly, 0.8μg of cDNA containing BK ZERO isoform vectors and 2.0μL of Lipofectamine 2000 were used during transfection. siRNA transfections we performed with 20pmol of miR-9 or miR-9/sponge and 1.0μl Lipofectamine 2000. Lipofectamine 2000 was diluted in Opti-MEM I Reduced Serum Medium with all transfections taking place in 24-well plates with a surface area of 2.0cm^2^ for each well.

**Fig 1:**
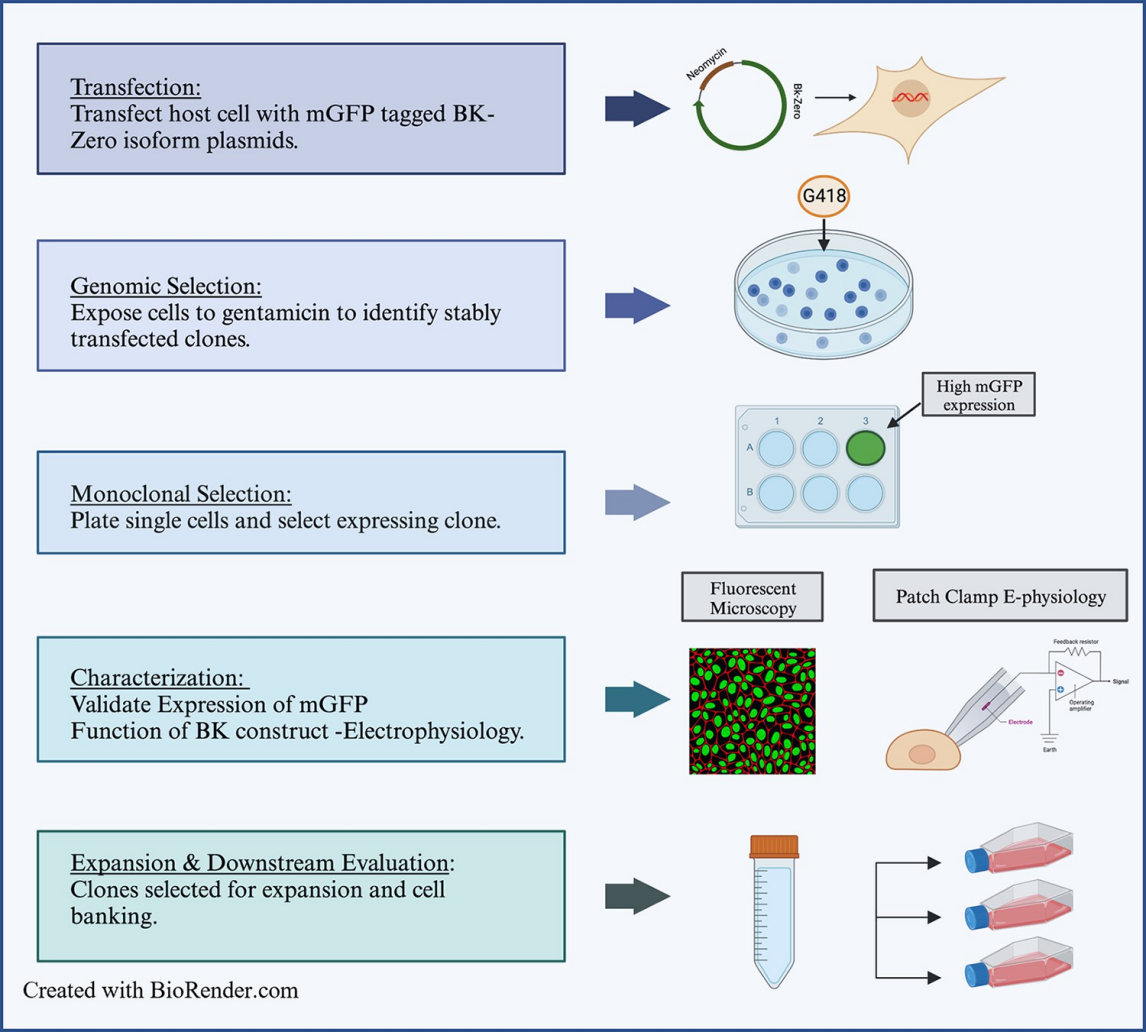
Procedures for establishing a BK-ZERO expressing HEK293 cell line. Schematic diagram summarizing the procedures used in the creation and validation of stability-transfected HEK293 cell lines expressing RES-, 2.1- and 2.2–3’UTR constructs. Generated with BioRender.

Vector constructs containing human BK channel α-subunit complexed with BK-mGFP variants (BK-ZERO RES, BK-ZERO 2.1, BK-ZERO 2.2) in Blue Heron’s plasmid cDNAs (S1–S3 Figs) were individually transfected into HEK293 cell cultures. After 16hrs of transfection, the transfection media was removed, and cells were washed with 1X PBS and placed in DMEM-supplemented media for 24hrs (Fig 1 and S1 File). Once transfected, the cultures proceeded to monoclonal selection to establish a stably transfected cell line for each variant. For miRNA experiments, 20pmol of either miR-9 sequence (Sigma-Aldrich/oligolearning, USA) or miR-9/sponge (Sigma-Aldrich/oligolearning with Mission Synthetic micro-Inhibitor (#cat. NCSTUD001) USA) were transiently transfected for 24hrs into the stably transfected BK variants of ZERO isoform cell lines.

### Monoclonal selection

After transfection with BK-mGFP variants, cells were washed 3 times with 1X PBS and placed in DMEM supplemented (without Pen/Strep) with 400μg/mL Gentamicin antibiotic G418 (Sigma Aldrich, A1720 Milwaukee, WI, USA). Cells were incubated for 10–14 days replacing media every 2–3 days. To create monoclonal cell lines, cells were plated onto 96-well by limited dilution as per the following protocol (https://www.addgene.org/protocols/limiting-dilution/). In brief, a 5cells/mL solution is prepared and used to seed a 96-well plate to ensure their sparsity and allow for the identification and isolation of monoclonal cell colonies. Cells were observed every other day to ensure a single cell source of isolated colony. When HEK293 cells with BK-mGFP variants grew to a confluency of 40%, cells were subcultured, to be authenticated and validated. Once the clone has been validated, cells are used for experimentation and/or stored in liquid nitrogen (Fig 1 and S1 File).

### Electrophysiology

Patch pipettes were made of borosilicate glass (VWR Micropipettes) with 6 – 8MΩ resistances. Solutions (in mM) were External (standard bath): 145 NaCl, 2.5 KCl, 10 HEPES, 2 CaCl_2_, 1 MgCl_2_, pH 7.5 **or** (ISO K^+^) 147 KCl, 10 HEPES, 2 CaCl_2_, 1 MgCl_2_, pH 7.5. Internal (pipette): 150 K-gluconate, 1 MgCl_2_, 10 HEPES, pH 7.4. Single-channel recordings were obtained in the “cell-attached” configuration and made with either standard bath or ISO K^+^ external solutions. When a standard bath was used, a correction for the effective applied voltage was made by estimating correction deviations using the leak currents. Dishes with monoclonal cell lines at a confluency of 30–50% were washed gently with standard bath solution (app. 20x dish volume, to ensure the removal of culture media) and let stand for ½ hr before use. The dish was then transferred to the recording chamber or, for the cases where high external K^+^ (ISO K^+^) was used, further washed with 3–5 dish volumes of high external K^+^ before testing. Recordings were started when a 1-3GΩ patch seal was established, and single-channel activity was observed. Data was acquired using a HEKA EPC10 patch-clamp amplifier controlled by a computer equipped with Patch Master acquisition software (HEKA Electronik). When using the standard whole-cell patch-clamp recording method, the membrane was subjected to various potentials for 0.5-1secs. Data were sampled at 25μs per sample and filtered at 10kHz low bandpass. Data analysis was performed with IgorPro graphing and curve fitting software (WaveMetrics). NP_o_ for each voltage was determined using the following:

$$NP_{o}=\frac{\sum_{i}iA_{i}}{\sum_{i}A_{i}}$$

where N is the number of observed channels (levels), i the i^th^ level number, and A_i_ the area of a Gaussian fit to the i^th^ level’s in all-points-histogram from each voltage. In the cases where the half-activation voltage (V_1/2_) was sought from NP_o_ versus voltage curves, the values were determined from sigmoidal fits assuming the following:

$${NP}_{o}=\frac{N}{1+e^{k(V_{\frac{1}{2}}-V)}}$$

where V_1/2_ is the half-activation voltage and V is the applied voltage. The V_1/2_’s were thus obtained from the half height of the sigmoidal fits (Table 1).

**Table 1:** Observed opening probability and Conductance of BK-ZERO channel isoforms. Given are voltages for NP_o_ half heights (V_1/2_, see Methods) and Conductances (in pS) for BK channel constructs. Values are the average for Control and same tested cells +ethanol (EtOH, at 20mM final bath concentration): one cell per dish was tested, thus, n represents single cells = single dishes. V_1/2_ shifts are defined as (EtOH V_1/2_)–(Control V_1/2_). All average values are for the set of paired N’s. Values of p were obtained from t-tests using paired data criteria. Errors (±) are given as SEM.

Construct	V_1/2_		V_1/2_ Shift	V_1/2_ p Value	Conductance		
	Control	EtOH	(EtOH V_1/2_) -(Control V_1/2_)		Control	EtOH	p Value
ZERO 2.2	-174.07 +/- 11.29	-152-61 +/-13.29	21.45 +/- 7.69	0.027	207.32 +/- 2.51	208.44 +/- 3.74	0.81
ZERO 2.1	-202.58 +/- 10.61	-168.64 +/- 29.18	39.93 +/- 7.22	0.00012	207.65 +/- 1.89	211.13 +/- 2.43	0.27
ZERO RES	-178.6 +/- 15.19	-155.60 +/- 5.07	23.01 +/- 5.67	0.015	214.82 +/- 4.09	215.81 +/- 2.97	0.87

### Immunocytochemistry

After electrophysiological confirmation of the presence of a functional BK-ZERO-mGFP stably transfected construct, monoclonal cell lines were grown on 3 distinct 100mm dishes (n = 3). Cell lines were then split into 35mm glass bottom dishes to a concentration of 100,000cells/mL (MATTEK Life Sciences, P35G-1.5-20-C MA, USA), creating 3 technical replicates (n) per biological replicates (N). The cells then underwent a transfection protocol with miR-9 or miR-9/sponge oligonucleotides. After the 48hr incubation time, cells were washed once with ice-cold 1X PBS, then fixated with Paraformaldehyde solution 4% in 1X PBS (PFA) (Santa Cruz Biotechnology, CAS 30525-89-4 TX, USA) for 1hr with gentle shaking. Subsequent steps were performed in the absence of light to prevent bleaching of GFP expression. Excess PFA was removed in three washes with ice-cold 1X PBS. Cells were then incubated with primary conjugated antibody of Cholera Toxin Subunit B Alexa Fluor 594 conjugate (C22842, Thermofisher, New York, NY, USA) added to label the lipid raft ganglioside, GM1 at 864ng/mL diluted in 1X PBS with 2mM Sodium Azide (Sigma Aldrich, S2002 S2002) for 1hr as a membrane marker. Membrane staining was followed by an additional 3 washes with 1X PBS. Samples were mounted with Aqueous Mounting Medium with DAPI (Santa Cruz Biotechnology, sc-24941 CA, USA) and covered with a square coverslip size 22 x 22–1 (Fisher Scientific,12-548-B USA).

### Fixed imaging and analysis

Confocal imaging was performed with Nikon Instruments A1 Confocal Laser Microscope using a 60X objective. Imaging of each experiment was done two weeks after mounting was complete and imaging parameters (Refractive Index = 1.51 and Numerical Aperture = 1.40) were maintained between treatments. Z-stacks through entire cells were taken, and mean intensity fluorescence and colocalization between fluorophores (Alexa Fluor 488 and 594) were analyzed by Nikon Software NIS Elements v5.30 –Software, Automated Measurements–Module.

### Statistics

BK expression in response to miR-9 was evaluated using an ordinary One-way ANOVA and Tukey post-hoc test. The confidence level was set to 0.05 (P-value). Error bars represent the standard error of the mean (SEM). The significance of these tests is displayed as: * p<0.05 (GraphPad Prism 10).

The protocol described in this peer-reviewed article is published on protocols.io (S1 File), https://dx.doi.org/10.17504/protocols.io.yxmvm3ek5l3p/v2 and is included for printing as S1 File with this article.

## Results and discussion

### Generation of stably transfected cell lines

**Fig 2:**
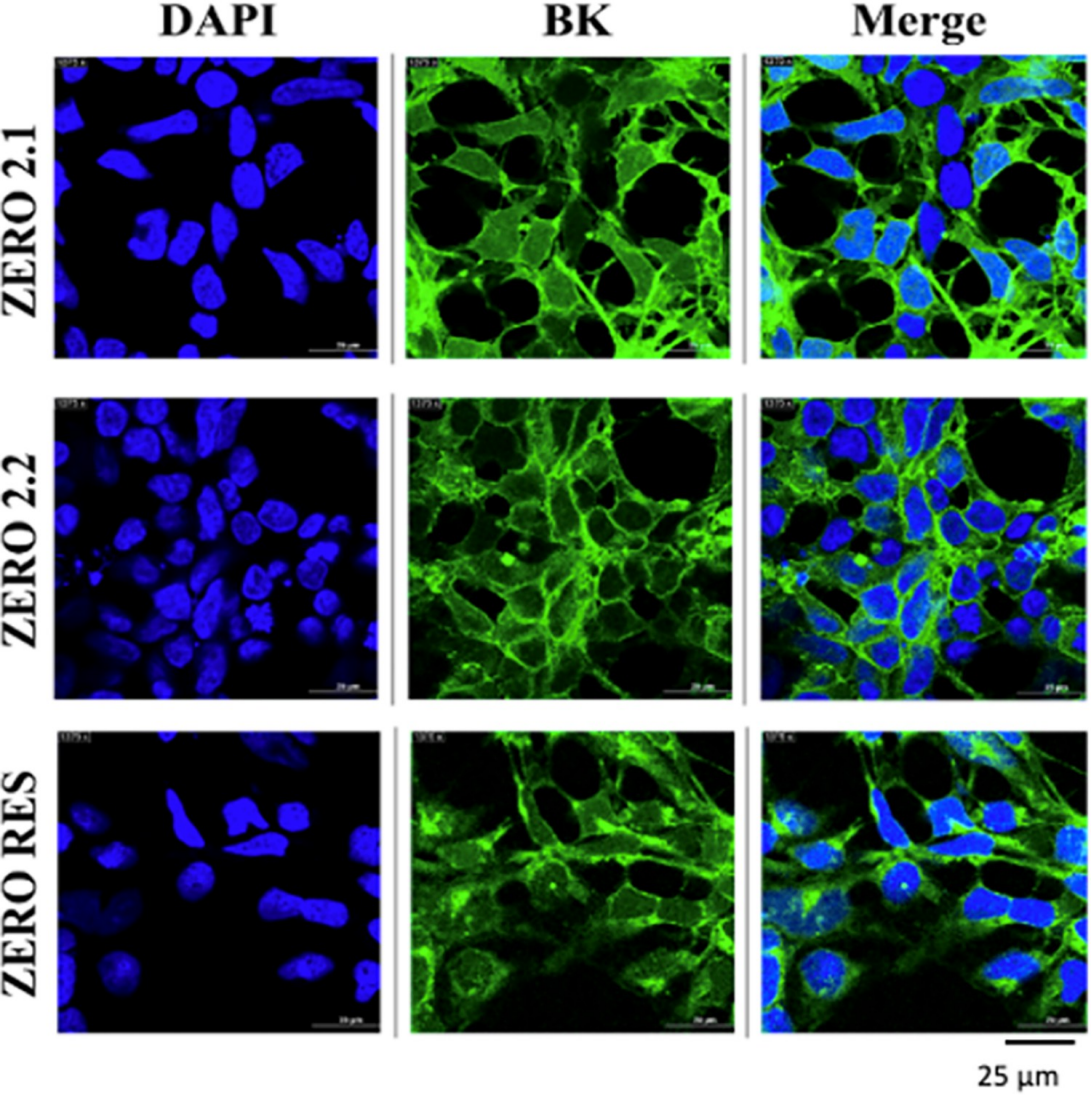
Validation of selected clones via GFP expression. Representative confocal images of stably transfected clones stained for DAPI nuclear staining (blue) and BK channel (green), and the merged images. Measure bar represents 25μm length.

To confirm the successful insertion of constructs expressing BK channel isoform with differing 3’UTR controlling miR-9 recognition sites, we performed immunocytochemistry. Transfection of the designed BK channel cDNA with GFP incorporated as a marker for the channel was successful in HEK293 cells (Fig 2). The selection of clones containing monoclonal cDNA incorporated into the genome resulted in individual clones. Validation of selected clones was collected via confocal images where there is a representation of the selected monoclonals of stably transfected ZERO expressing either 2.1, 2.2, or no 3’UTR control (RES) isoforms. Confocal images were collected from nuclear staining (DAPI) and GFP-expression from each BK channel isoform. The creation of cell lines with BK isoforms, ZERO (alcohol-regulated exon) will provide a better understanding of the development of BK channel-mediated molecular tolerance and the mechanism behind BK channel expression and redistribution. One advantage of using HEK293 cells is that they express both the Wnt/ß-catenin signaling and miR-9 pathways which are the focus of our study. This makes it possible to study neuronal regulatory mechanisms that play a key role in alcohol regulation with a reliable, low-maintenance cell culture that expresses human equivalent regulatory factors readily compatible with translational studies [22, 30, 31].

#### Limitations

The experimental system is limited in how it can replicate a neuronal environment as they are grown in the absence of glia and other neuronal supporting cells which can impact their properties. Furthermore, the lack of synaptic connectivity needs to be taken into consideration for future studies given ion channel expression and distribution has been shown to be linked to activity-dependent processes not currently under the scope of this study.

### Functionality of transfected BK- ZERO channels

**Fig 3:**
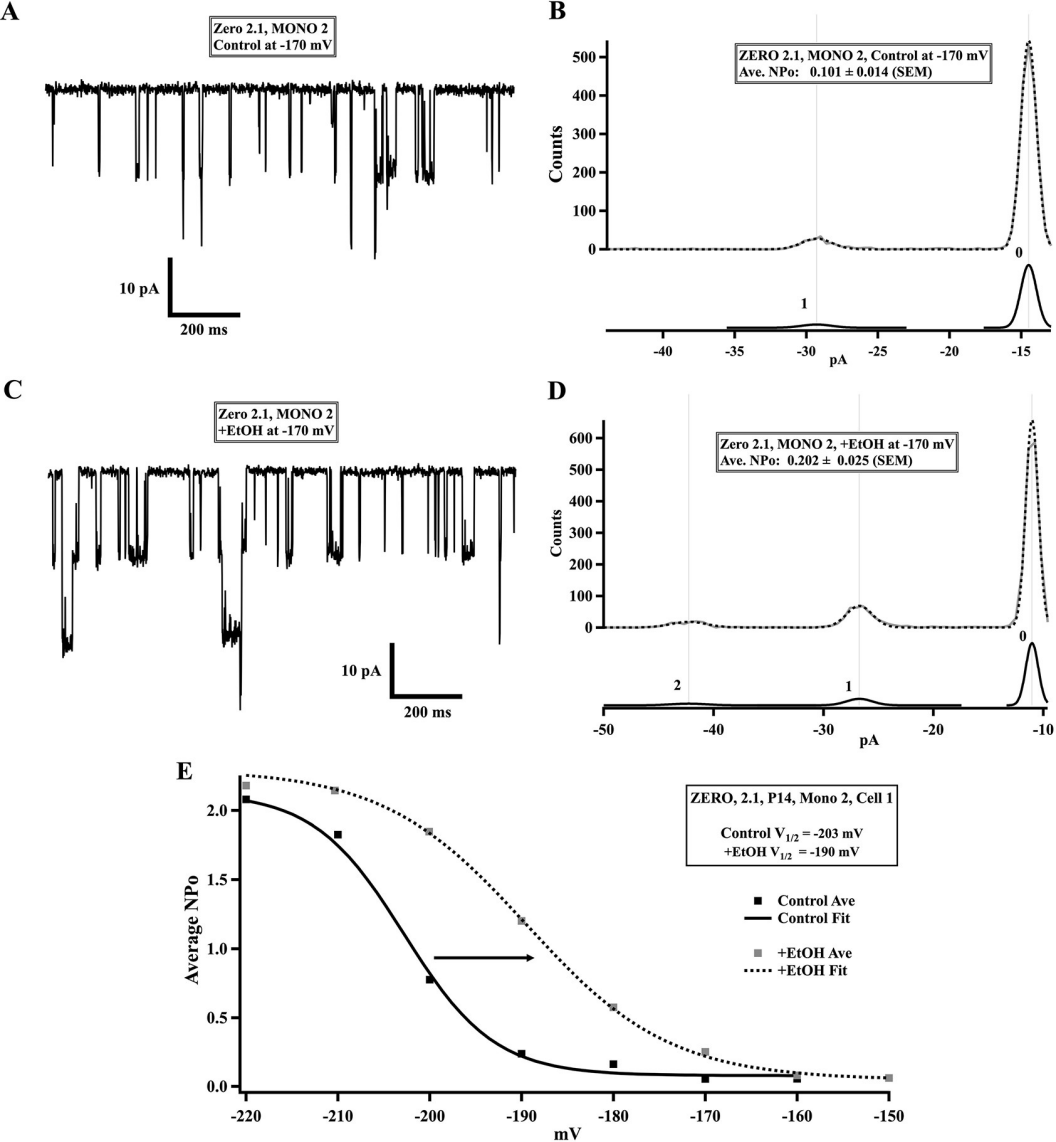
Functional characterization of expressed ZERO 2.1 –BK channel isoform channel. A, Representative single channel recording from the cell-attached mode under control conditions. B, Histogram of all points counts under control conditions (all-points histogram) for one applied voltage (-170mV): dashed black trace is the algorithm’s predicted curve using Gaussian fits (solid black traces with numbered peaks, below, 0 being the closed state counts, 1 the 1^st^ state, etc.) and gray trace is the raw data curve. C, Representative single-channel traces after acute 20mM EtOH exposure on the same cell. D, All-points histogram under EtOH treatment. NP_o_ is determined from the area of the Gaussian fits (see methods). E, Plot of NP_o_ averages at different applied voltages (square dots) for control (black) and +EtOH (gray), with corresponding sigmoidal fits (solid and dashed curves, respectively) for one cell. Each square dot is the average of 5–10 runs on the same patch. The V_1/2_ is determined from the voltage value at half-height of each (see also methods). Application of EtOH shifts the V_1/2_ to less negative values (arrow). In the cell-attached mode, this translates as voltages that would depolarize the patched membrane less to obtain the same NP_o_’s as with controls.

Electrophysiology was used to determine if transfected channels expressed their expected function, while still being at the surface of transfected live cells This was done by establishing the conductance (200-220pS), the NP_o_-V relationship (sigmoidal, see “Electrophysiology section), and the acute sensitivity to alcohol 20mM [30–32]. The ZERO isoform increases its activity (NP_o_) in response to acute 20mM alcohol treatment (Fig 3). However, due to the NP_o_ form function (NP_o_ curves), the increases caused by ethanol are not the same for different voltages. The ethanol-related changes in NP_o_ are therefore better represented as shifts in the NP_o_-V curves, where increases in activity would translate as shifts to more negative membrane potentials (i.e., more hyperpolarized at half-heights, V_1/2_). The sense of these ethanol-caused shifts was found to be consistent, despite the results for controls having a large variability (perhaps due to synergies within live cells).

### Characterization of BK channel surface expression in response to miR-9 and miR-9/Sponge

**Fig 4:**
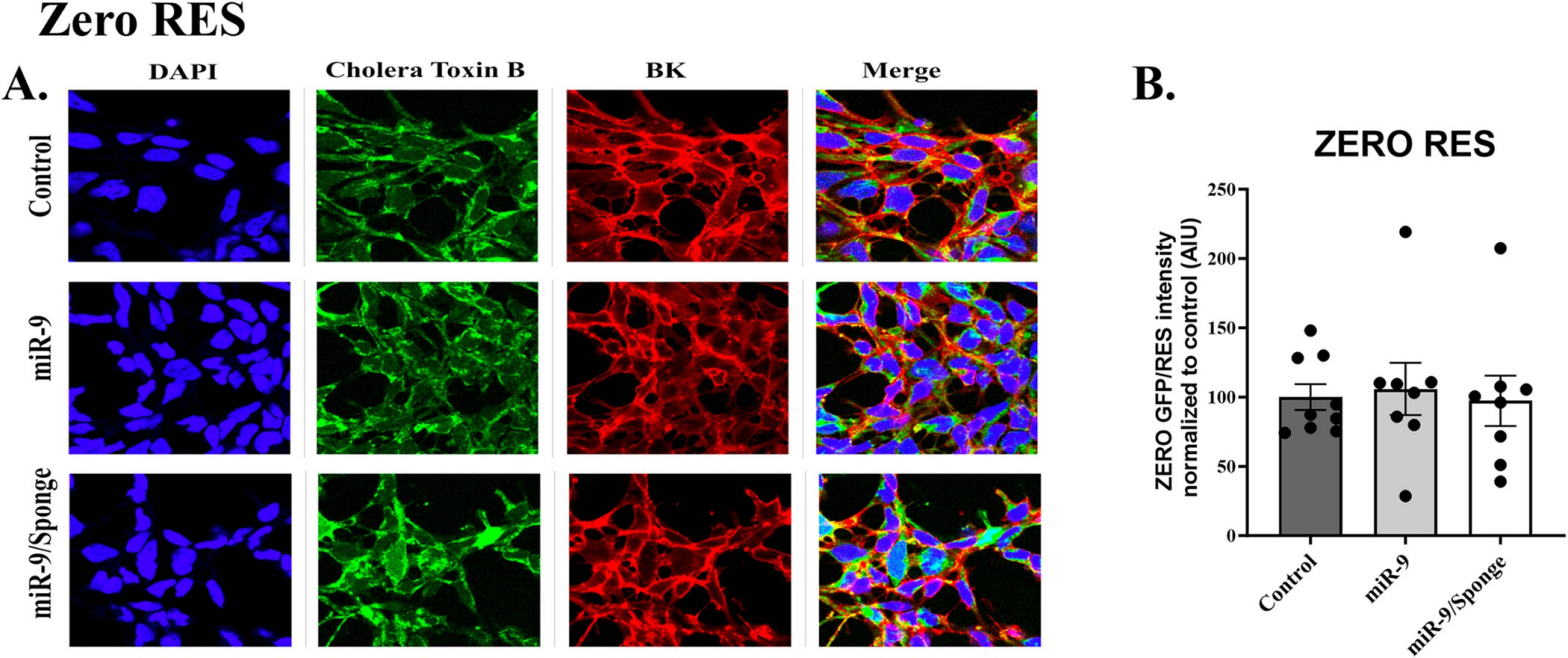
Characterization of BK channel surface expression in response to miR-9 and miR-9/Sponge of ZERO constructs differing in their 3’UTR sequence. ***A*.** Representative confocal images of stably transfected ZERO cell line 2.1, for DAPI (blue), Conjugated Cholera Toxin B (ALEXA Flour 594, red) as a membrane marker, and GFP-expression of BK channel constructs (green) and merged images. Measure bar length represents 10μm. ***B*.** Bar graph quantification of arbitrary intensity units (AUI) of GFP-488 fluorescence of BK channel expression under control, miR-9 and miR-9/Sponge treatment. Data presented as a mean and S.E.M.

**Fig 5:**
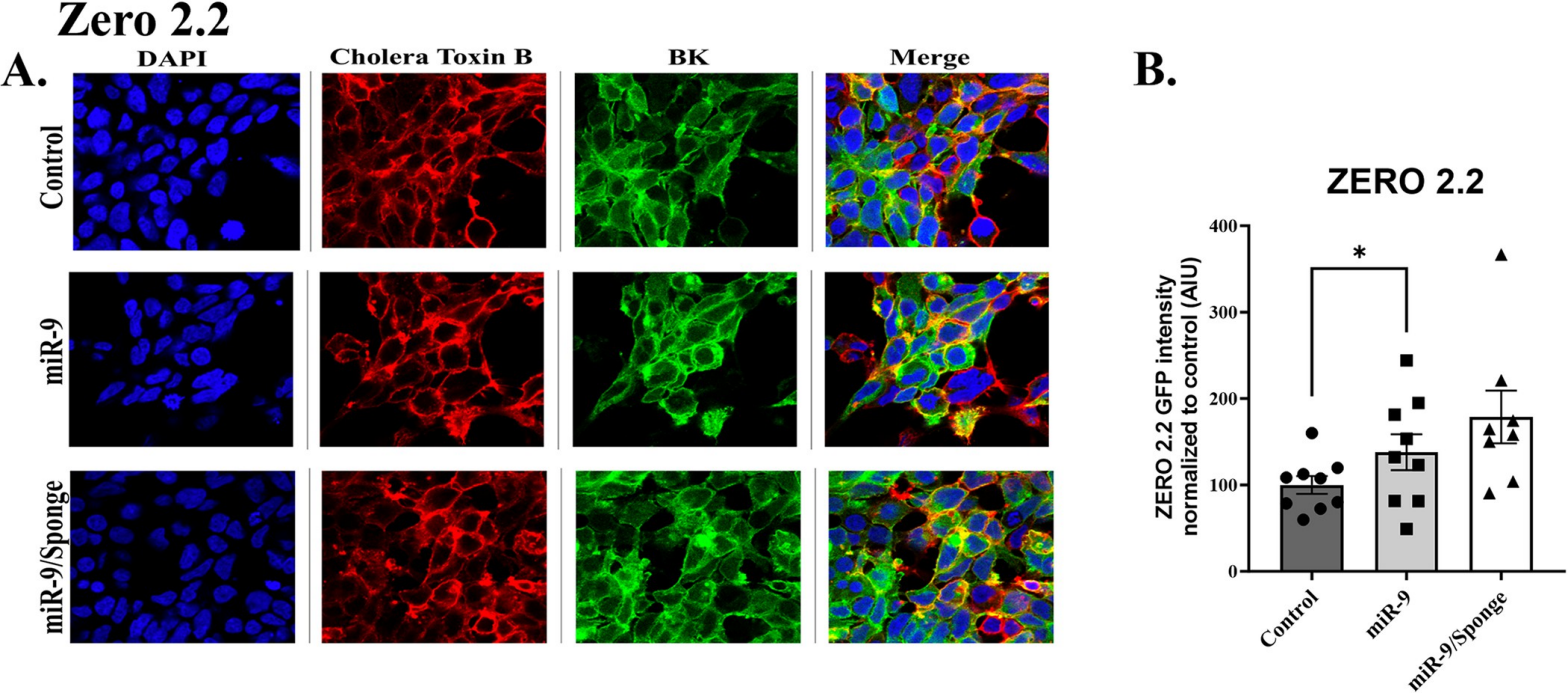
Characterization of BK channel surface expression in response to miR-9 and miR-9/Sponge of ZERO constructs differing in their 3’UTR sequence. ***A*.** Representative confocal images of stably transfected ZERO cell line 2.2, for DAPI (blue), Cholera Toxin B (red) as a membrane marker, and GFP-expression of BK channel constructs (green) and merged images. Measure bar length represents 10μm. ***B*.** Bar graph quantification of arbitrary intensity units (AUI) of GFP-488 fluorescence of BK channel expression under control, miR-9 and miR-9/Sponge treatment. Asterisks represent a statistically significant difference, * p<0.05. Data presented as a mean and S.E.M.

**Fig 6:**
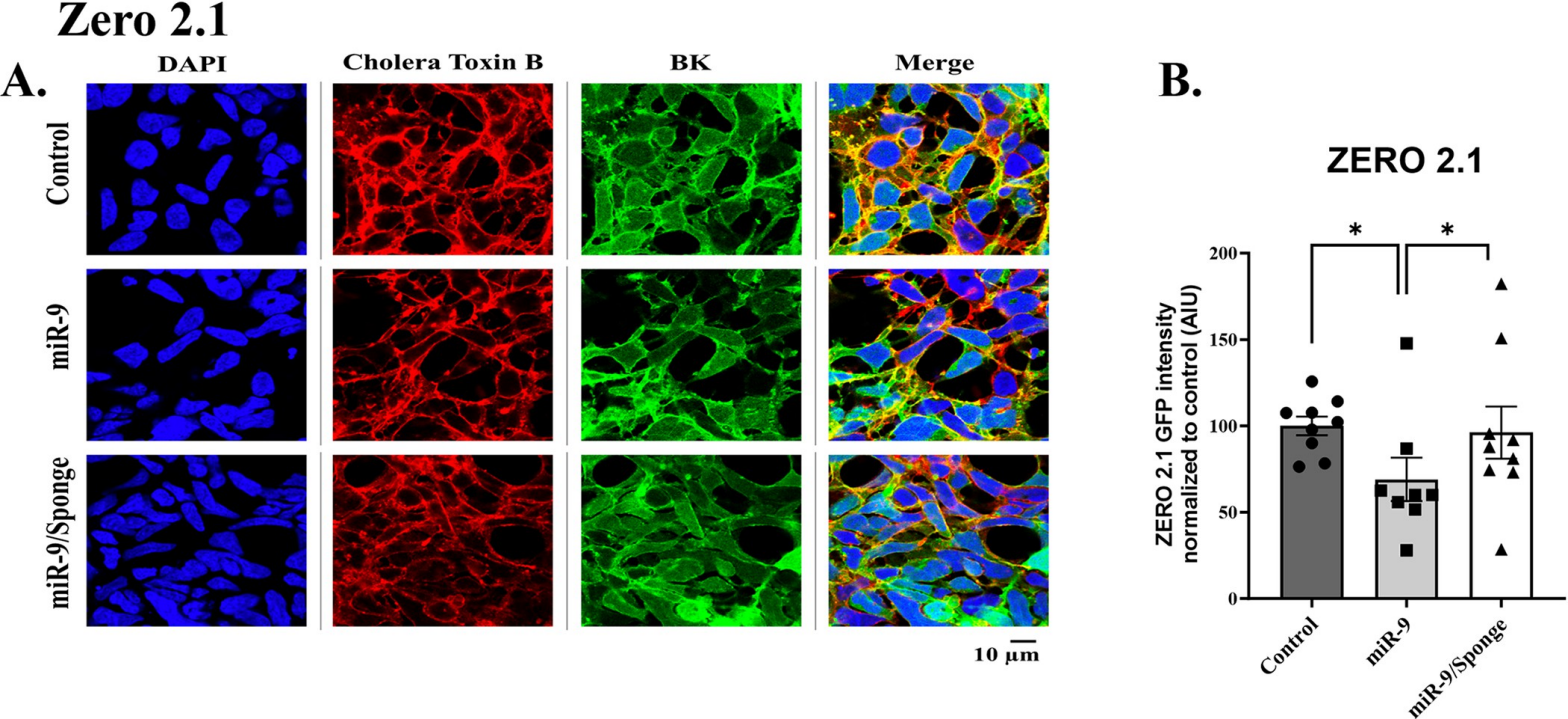
Characterization of BK channel surface expression in response to miR-9 and miR-9/Sponge of ZERO constructs differing in their 3’UTR sequence. ***A*.** Representative confocal images of stably transfected ZERO cell line RES, for DAPI (blue), Cholera Toxin B (red) as a membrane marker, and GFP-expression of BK channel constructs (green) and merged images. Measure bar length represents 10μm. ***B*.** Bar graph quantification of arbitrary intensity units (AUI) of GFP-488 fluorescence of BK channel expression under control, miR-9 and miR-9/Sponge treatment. Asterisks represent a statistically significant difference, * p<0.05. Data presented as a mean and S.E.M.

Reactivity to miR-9 was assessed by analyzing the surface expression of BK channels using immunocytochemistry. Bk channel was identified with stably transfected GFP, Cell membranes were identified using Cholera Toxin Subunit B (Recombinant), Alexa Fluor™ 594 Conjugate (Invitrogen™ C22842, Waltham MA, USA), the nucleus was stained with DAPI and merged images were also constructed and presented (Figs 4–6A). GFP expression analysis for BK: ZERO RES, ZERO 2.1, and ZERO 2.2 channel isoforms during the upregulation of miR-9 were analyzed by fluorescence mean intensity using Nikon Software NIS Elements v5.30 –Software, Automated Measurements–Module. To confirm reactivity to miR-9 we performed an ordinary One-way ANOVA of mean intensity values for Control, miR-9, and miR-9 sponge-treated groups for each of our transfected lines (ZERO RES, ZERO 2.1, and ZERO 2.2). Analysis of ZERO 2.1 expressing cells revealed a significant difference between treatment groups: F (2, 6) = 10.62, p = 0.0107, Tukey Post Hoc test revealed a significant reduction in the miR-9 treatment group when compared to both control (31% reduction ± 12.61, p = 0.0132), and miR-9 sponge group (27% reduction ± 15.04, p = 0.0230) (Fig 4). Ordinary One-way ANOVA of ZERO 2.2 expressing cells also revealed a significant difference between treatment groups: F (2, 6) = 6.256 p = 0.0341, Tukey Post Hoc test revealed a significant increase between control and miR-9 sponge groups (77% increase ± 19.28, p = 0.0284) (Fig 5). ANOVA of RES showed no significant differences (Fig 6). A biological N of 3 was used for all groups data is presented as mean and SEM normalized to control. The downregulation of BK by miR-9 in ZERO 2.1 confirms its reactivity to the compound and reveals miR-9’s role in the translational downregulation of the BK channel isoform where a complementary sequence for the microRNA is found in the 3’UTR.

## Conclusion

Our results confirm the successful stable transfection of HEK293 cells with BK constructs containing either ZERO RES, ZERO 2.1, or ZERO 2.2 3’UTR sequences. We validated the functional expression of the channel using electrophysiological recordings to confirm typical conductance and voltage-current relationship. We further identified characteristic increases in open channel probability in response to alcohol corroborating ZERO BK channel acute alcohol sensitivity. Finally, we exposed stably transfected cell lines to miR-9 and quantified overall BK channel protein expression by quantifying GFP fluorescence. Results showed HEK293 cell lines expressing 2.1 3’UTR miR-9 recognition sites had significantly less expression of BK channels in the presence of miR-9, as opposed to cell lines containing scrambled miR-9 recognition sequences or no miR-9 recognition sites, such as ZERO 2.2 and ZERO RES. These transfected cell lines are thus, suitable as a model to study alcohol’s role in regulation of BK surface expression as a function of miR-9 regulation. These cellular tools have the potential to provide insights into the molecular underpinnings of alcoholism and could improve our overall understanding of the role of neuroplasticity and epigenetics in addiction.

## Supporting information

S1 File https://dx.doi.org/10.17504/protocols.io.yxmvm3ek5l3p/v2. (PDF)

S1 Fig BK-ZERO 2.1 construct sequence. Sequence for transfected construct containing the 2.1 3’UTR variation of BK channel detailing several key portions. Yellow: miR-9 MRE, Dark Blue: 3’UTR 2.1 sequence, Grey: poly-A tail, Cian: Coding sequence for BK channel, Green: sequence of mouse GFP, Magenta: restriction enzyme sites (Blue Heron Biotech, LLC).
(TIFF)

S2 Fig BK-ZERO 2.2 construct sequence. Sequence for transfected construct containing the 2.2 3’UTR variation of BK channel detailing several key portions. Dark Blue: 3’UTR 2.1 sequence, Grey: poly-A tail, Cian: Coding sequence for BK channel, Green: sequence of mouse GFP, Magenta: restriction enzyme sites (Blue Heron Biotech, LLC).
(TIFF)

S3 Fig BK-ZERO RES construct sequence. Sequence for transfected construct containing the no 3’UTR detailing several key portions. Grey: poly-A tail, Cian: Coding sequence for BK channel, Green: sequence of mouse GFP, Magenta: restriction enzyme sites (Blue Heron Biotech, LLC).
(TIFF)
