# Supplementary material for: BK ZERO isoform HEK293 stably transfected cell lines differing 3’UTRs to assess miR-9 regulation
Source: PLoS One. 2024 Mar 19;19(3):e0298966. doi: 10.1371/journal.pone.0298966 (PMC10950231; doi:10.1371/journal.pone.0298966)
Supplement: S1 File — (PDF) [file pone.0298966.s001.pdf]

Transfection and validation of BK channel expressing HEK-293 cells for the study of miR-9 regulation. V.2

Katherine Cordero Padilla[1,2,3], Gerardo L. Alvarado Monefledt[4,5], Adriel Guevarez-Galan[1,2,6], Hector G Marrero Hernandez[2], Mario E. Lloret-Torres[1,2], Cristina Velazquez Marrero[1,2]

[1]University of Puerto Rico Medical Sciences Campus, Department of Anatomy and Neurobiology, San Juan, Puerto Rico 00936;
[2]Institute of Neurobiology, University of Puerto Rico Medical Sciences Campus, San Juan, Puerto Rico 00901;
[3]Windsor University School of Medicine, St. Kitts, West Indies;
[4]University of Puerto Rico, Cayey Campus Department of Biology, Cayey, Puerto Rico 00736;
[5]Samuel Merritt University, Oakland, California, 94609;
[6]University of Puerto Rico, Bayamón Campus Department of Biology, Bayamón Puerto Rico 00959.

Mario E. Lloret-Torres

VERSION 2

NOV 29, 2023

OPEN ACCESS

DOI:
dx.doi.org/10.17504/protocols.io.yxmvm3ek5l3p/v2

Protocol Citation: Katherine Cordero Padilla, Gerardo L. Alvarado Monefledt, Adriel Guevarez-Galan, Hector G Marrero Hernandez, Mario E. Lloret-Torres, Cristina Velazquez Marrero 2023. Transfection and validation of BK channel expressing HEK-293 cells for the study of miR-9 regulation.. **protocols.io** https://dx.doi.org/10.17504/protocols.io.yxmvm3ek5l3p/v2
Version created by Mario E. Lloret-Torres

License: This is an open access protocol distributed under the terms of the Creative Commons Attribution License, which permits unrestricted use, distribution, and reproduction in any medium, provided the original author and source are credited

DISCLAIMER

There are no
competing interests financial or non-financial, professional, or personal.

Protocol status: Working
We use this protocol and it's working

Created: Nov 27, 2023

Last Modified: Nov 29, 2023

PROTOCOL integer ID: 91478

Keywords: BK channel, STREX, ZERO, MiR-9, ICC, HEK293, 3’UTR, EtOH.

Funders Acknowledgement:
NIAAA
Grant ID: NIH R01AA027808 (CVM)
NIH-COBRE
Grant ID: 2P20GM103642-08

ABSTRACT

Research has identified the large conductance voltage- and calcium-activated potassium channel (BK) as a key regulator of neuronal excitability genetically associated to behavioral alcohol tolerance. Sensitivity to ethanol at the molecular level is characterized by acute potentiation of channel activity. BK isoforms show variations in alcohol sensitivity and are differentially distributed on the plasma membrane surface in response to prolonged exposure. MicroRNA (MiRNA) targeting of alcohol-sensitive isoforms coupled with active internalization of BK channels in response to ethanol are believed to be key in establishing homeostatic adaptations that produce persistent changes within the plasma membrane of neurons. In fact, microRNA 9 (miR-9) upregulated expression is a key event in persistent alcohol tolerance mediating acute EtOH desensitization of BK channels. The exact nature of these interactions remains a current topic of discussion. To further study the effects of miR-9 on the expression and distribution of BK channel isoforms we designed an experimental model by transfecting human BK channel isoforms ZERO heterologous constructs in human embryonic kidney cells 293 (HEK293) cells respectively expressing 2.1 (miR-9 responsive), 2.2 (unresponsive) and control (no sequence) 3’untranslated region (3’UTR) miRNA recognition sites. We used imaging techniques to characterize the stably transfected monoclonal cell lines, and electrophysiology to validate channel activity. Finally, we used immunocytochemistry to validate isoform responsiveness to miR-9. Our findings suggest the cell lines were successfully transfected to express either the 2.1 or 2.2 version of ZERO. Patch clamp recordings confirm that these channels retain their functionality and immunohistochemistry shows differential responses to miR-9, making these cells viable for use in future alcohol dependence studies.

GUIDELINES

All cell culture procedures were performed inside a biological hood using a sterile cell culture technique. HEK-293 is a cell line exhibiting epithelial morphology that was isolated from the kidney of a human embryo commercially available through ATCC (American Type Culture Collection) a nonprofit, global biological resource center.

MATERIALS

Cell transfection/Monoclonal selection

- Human Embryonic Kidney Cells (HEK293) (ATCC, CRL-1573, VA, USA)

- OptiMEM (ThermoFisher, A41248-02, NY, USA)

- Lipofectamine 2000 (11668027 ThermoFisher, Lithuania)

- Blue Heron's plasmid cDNAs:

Protocol: Genemaker® Gene Synthesis Platform from Blue Heron, LLC

The company uses Genemaker® which is a fully automated gene synthesis platform designed to facilitate the entire gene synthesis process. The process began with inputting the desired gene sequence into the website ordering page. The company proceeded to optimize the gene build based on the entered sequence using a proprietary algorithm. The optimization aimed to balance complexity reduction and protein expression enhancement. Once the Oligonucleotides are synthesized the are amplified via PCR synthesis techniques, utilizing both solution and solid substrate technologies. The synthesized genes are then cloned into the vector of choice. The cloned gene is sequence verified to ensure 100% accuracy and ship to us.

Project #	Vector Name	Common Name
509802	CCN139060	ZERO RES
509801	CCN134655	ZERO 2.2
509797	CCN134654	ZERO 2.1
509800	CW101512	STREX RES
509802	CW101512PA-BKalpha3UTR2	STREX 2.2
509799	CW101512PA-BKalpha3UTR	STREX 2.1

Table. Summary of Transfected Plasmids.

- miR-9 sequence and mimic (Sigma-Aldrich/oligolearning, USA)
- miR-9/sponge (Sigma-Aldrich/oligolearning with Mission Synthetic micro-Inhibitor (#cat. NCSTUD001) USA)
- Dulbecco's modified Eagle's medium (DMEM) (D5796, Milwaukee, WI, USA)

- 10% fetal bovine serum (FBS) (F-4135), 2.52 mM

- Penicillin/Streptomycin (Pen/Strep)(P4333, Saint Louis, MO, USA)

- Na-pyruvate (Sigma-Aldrich, S8636 Milwaukee, WI, USA)

- Tripsin-EDTA 10X (ThermoFisher, 15400-054 New York, NY, USA)
- Phosphate Buffered Saline (PBS) (Sigma Aldrich, 806552 Milwaukee, WI, USA)
- Trypan Blue dye (BioRad, 64315131, UK)
- G418 antibiotic (Sigma Aldrich, A1720 Milwaukee, WI, USA)
- 35mm petri dish (Corning 353001, NY, USA)
- Counting slides (BioRad, 64302432, USA)
- 96-well plates (Millipore SIGMA, CLS3922)

Electrophysiology

- Borosilicate glass (VWR Micropipettes)
- NaCl – sodium chloride
- KCl – potassium chloride
- HEPES - N-2-hydroxyethylpiperazine-N-2-ethane sulfonic acid
- $CaCl_2$ - calcium chloride
- $MgCl_2$ - magnesium chloride
- 150 K-gluconate

Immunocytochemistry

- 100mm dishes (Millipore sigma, Z358762)
- 35mm glass bottom dishes (MATTEK Life Sciences, P35G-1.5-20-C MA, USA)
- miR-9 or pBabe-puro-miR-9 sponge (Addgene, 2504).
- Paraformaldehyde solution 4% in PBS (PFA) (Santa Cruz Biotechnology, CAS 30525-89-4 TX, USA)
- Cholera Toxin Subunit B Alexa Fluor 594 conjugate (C22842, ThermoFisher, New York, NY, USA)
- Sodium Azide (Sigma Aldrich, S2002 S2002)
- Aqueous Mounting Medium with DAPI (Santa Cruz Biotechnology, sc-24941 CA, USA)
- Square coverslip size 22 x 22 -1 (Fisher Scientific,12-548-B USA)

Equipment

- Sanyo CO2 incubator MCO-18AC (Sanyo Electric Co., Japan)
- Purifier Biological Safety Cabinet Class//typeA2 (LABCON Co.)
- Vacuum pump p-79202-00 (Cole Parmer Instrument Co.)
- HEKA EPC10 amplifier controlled with PATCHMASTER program (all by HEKA

Elektronik, Dr.
Schulze GmbH, Wiesenstraße 71, D-67466 Lambrecht/Pfalz, Germany).

SAFETY WARNINGS

Please see SDS (Safety Data Sheet) for hazards and safety warnings.

Cell Culture

1 Thaw cells and then plate them as follows:

1.1 Resuspend in 1mL of modified DMEM media composed of Dulbecco's modified Eagle's medium (D5796, Milwaukee, WI, USA) with 10% fetal bovine serum (FBS), 2.52mM HEPES; 10 units and 10µg/mL of Penicillin/Streptomycin (Pen/Strep), and 1mM Na-pyruvate.

1.2 Pipette 20µL of the resuspended solution onto 35mm petri dishes (surface area of 8.8cm^2).

1.3 Flood with DMEM up to a final volume of 2mL.

2 Place cells in a Sanyo CO_2 incubator (37.0^{o}C/5% CO_2 level) and allow them to reach full confluency replacing the media every 3-5 days.

3 Once the cells reach 70-90% confluency suction media from the plates and add Trypsin-EDTA 1:10 diluted in 1X PBS to solubilize the cells. Apply gentle shaking to the plates to allow for the separation of the cells from the plate. Add a volume of DMEM to the plate equal to the added volume of trypsin to prevent further cell lysis.

4 Pipette suspended cells onto 10mL tubes.

5 Remove 10mL tubes from the chemical hood and centrifuge at 1,380g for 60 seconds.

6 Place the tubes inside the chemical hood and aspirate the supernatant.

7 Resuspend in 1mL of DMEM.

8 Extract 10µL of each tube and mix with equal volume of Trypan Blue Dye.

9 Add a single droplet of the Trypan Blue mixture to a counting slide and place in an automated cell counter.

10 While the cells are being counted, add an additional 9mL of modified DMEM to the tube and gently resuspend, for a total volume of 10mL.

11 Once counted add the appropriate seeding density (0.3x10^6 cells) to the 35mm petri dishes and place

them in the incubator and allow them to reach 70-90% confluence.

Transfection (BK ZERO isoform cDNA)

12 Reseed cells onto a 24-well plate and incubate until they reach 70-90% confluence.

13 Once cells reach 70-90% confluence remove the plates from the incubator and place them in the cell culture hood.

14 Wash the plates 3 times with 1X PBS for 30 seconds. Gently swirl between washes.

15 Add serum-deprived DMEM (no FBS) to the plates and place them in the incubator for 24 hours.

16 After incubation prepare a 100µL solution of Opti-MEM reduced serum media containing 0.8µg of cDNA BK ZERO isoform vector and 2.0µL of Lipofectamine 2000 (11668027, Thermofisher, NY, USA).

17 Incubate mixture for 5 minutes at room temperature before use.

18 Add the mixture to each well in the 24-well plate and place them in the incubator for 16 hours to allow for transfection to occur.

19 After the incubation period, completely exchange transfection media with DMEM.

20 Wash 3 additional times with 1X PBS and place in modified DMEM for 24 hours in the incubator.

Transfection (miR-9 and miR-9 sponge)

21 Reseed cells onto a 24-well plate and incubate until they reach 70-90% confluence.

22 Once cells reach 70-90% confluence remove the dishes from the incubator and place them in the cell culture hood.

23 Wash the plates 3 times with 1X PBS for 30 seconds. Gently swirl between washes.

24 Add serum-deprived DMEM (no FBS) to the plates and place them in the incubator for 24 hours.

25 After incubation prepare a 100µL solution of Opti-MEM reduced serum media containing 0.8µg of cDNA BK ZERO isoform vector and 2.0µL of Lipofectamine 2000 (11668027, Thermofisher, NY, USA).

26 Incubate mixture for 5 minutes at room temperature before use.

27 Add the mixture to each well of the 24-well plate and place them in the incubator for 24 hours to allow for transfection to occur.

28 After the incubation period, completely replace the transfection media with DMEM the reduced.

29 Wash 3 additional times with 1X PBS and place in modified DMEM for 24 hours in the incubator.

Monoclonal Selection

30 After transfection, of BK ZERO constructs, remove cells from the incubator and wash 3 times (30 secs each) with 1X PBS. Add DMEM supplemented with 400µg/mL of G418 (Gentamicin)(Sigma Aldrich, A1720 Milwaukee, WI, USA) antibiotic.

31 Replace half of the media volume with modified DMEM every 2-3 days for 10-14 days or until visually resistant colonies are visible.

32 Transfer cells from the 24-well plate (as described in the cell culture section) and plate onto 96-well plate by limited dilution (to create monoclonal cell lines) as follows:

32.1 In a 96-well plate pipette 12µL of cell culture media into all except A1.

32.2 Pipette 200µL of the cell suspension into well A1.

32.3 Pipette 100µL from well A1 to A2 and gently resuspend gently.

32.4 Repeat dilutions from A2 to A3 and subsequently throughout the entire row.

32.5 Then Pipette 100µL from all wells on Row A too wells on row B and resuspend gently.

32.6 Repeat this process for pair of rows (B-C, C-D...).

32.7 Add 100µL of media to each well.

32.8 Incubate for 4 to 5 days.

33 Observe daily to ensure colonies were formed by a single cell.

34 Identify wells containing colonies from single-cell origin. Allow these colonies to reach 40% confluence. Subculture these colonies individually for validation of construct genomic insertion.

Electrophysiology

35 Stably transfected cells are plated in 35mm dishes and allowed to reach 30-50% confluence. Prior to experiments, dishes are gently washed 3 times with 1X PBS to remove any remaining fetal bovine serum from media; and then placed in Normal Locke's solution (mM):145 NaCl, 2.5 KCl, 10 HEPES, 2 $CaCl_2$, 1 $MgCl_2$, pH 7.5.

36 Allow plates to stand 30 minutes before use.

37 Single-channel recordings are obtained in the cell-attached patch clamp mode under the following conditions:

37.1 Internal (pipette)solution (mM): 150 k-gluconate, 1 $MgCl_2$, 10 HEPES, PH 7.4.

37.2 External bath solution (mM): 145 NaCl, 2.5 KCl, 10 HEPES, 2 $CaCl_2$, 1 $MgCl_2$, pH 7.5.

or

ISO K^+: 147 KCL, 10 HEPES, 2 $CaCl_2$, pH 7.5.

38 Transfer plates to the recording chamber and wash dishes with ISO K^+ solution 3 to 5 times (30 secs each).

39 Carefully create a patch seal of 1-3GΩ and begin recording.

40 When using the standard bath, correct for the effective applied voltage by estimating correction deviations using the leak currents during cell-attached recordings.

41 Aquire Data using a HEKA EPC10 patch-clamp amplifier controlled by a Macintosh-based computer system equipped with Patch Master acquisition software.

42 Sample Data at 25µs per sample and filtered at 10kHz low bandpass.

Electrophysiology Data Analysis

43 Perform data analysis with IgorPro graphing and curve fitting software as follows:

43.1 Determine NP_o for each voltage using the following:

$$NPo = (\sum_i (iAi))/(\sum_i (Ai))$$

where N is the number of observed channels (levels), i the ith level number, and A_i the area of a Gaussian fit to the ith level's in all-points-histogram from each voltage.

44 When seeking the half-action voltage ($V_{1/2}$) from NP_o versus voltage curves, determine the values from sigmoidal fits assuming the following:

$$NPo = N/(1 + e^(k(V(1/2) - V))$$

where $V_{1/2}$ is the half-action voltage and V the applied voltage. The $V_{1/2}$'s were thus, obtained from the half height of the sigmoidal fits.

Immunocytohemistry (ICC)

45 For ICC cells were plated in 35mm glass bottom petri dishes (MATTEK Life Sciences, P35G-1.5-20-C MA, USA).

46 Cells were allowed to reach 50-60% confluence and then removed from the incubator and then wash 3 times with 1X PBS (30 secs each).

47 Cell were fixed by placing plates in a 4% paraformaldehyde (PFA) solution (Santa Cruz Biotechnology, CAS 30525-89-4 TX, USA) for 1 hour while gently rocking.

48 Excess PFA was removed with 3 washes using ice-cold 1X PBS.

49 Cells were first incubated for 1 hour with Cholera Toxin B Alexa Fluor 594 conjugate (CtB) antibody(C22842,Thermofisher, New York, NY, USA) 864ng/mL to label GM1.

50 Wash 3 additional times with 1X PBS.

51 Following the final wash, remove all 1X PBS.

52 Add Fluoromount-G™ Mounting Medium, with DAPI 00-4959-52 (Thermo Fisher) to the dishes prior to placing a cover slip, Premium Cover Glass 12-548-B (Fisher Scientific USA).

53 Image using Nikon Instruments A1 Confocal Laser Microscope with 60X objective (Refractive Index=1.51 and Numerical Aperture=1.40) (Maintain imaging parameters between treatments).

54 Analyze Mean intensity fluorescence and colocalization by Nikon Software NIS Elements v5.30 – Software, Automated Measurements – Module.
