# Supplementary figures and images for: BK ZERO isoform HEK293 stably transfected cell lines differing 3’UTRs to assess miR-9 regulation
Source: PLoS One. 2024 Mar 19;19(3):e0298966. doi: 10.1371/journal.pone.0298966 (PMC10950231; doi:10.1371/journal.pone.0298966)

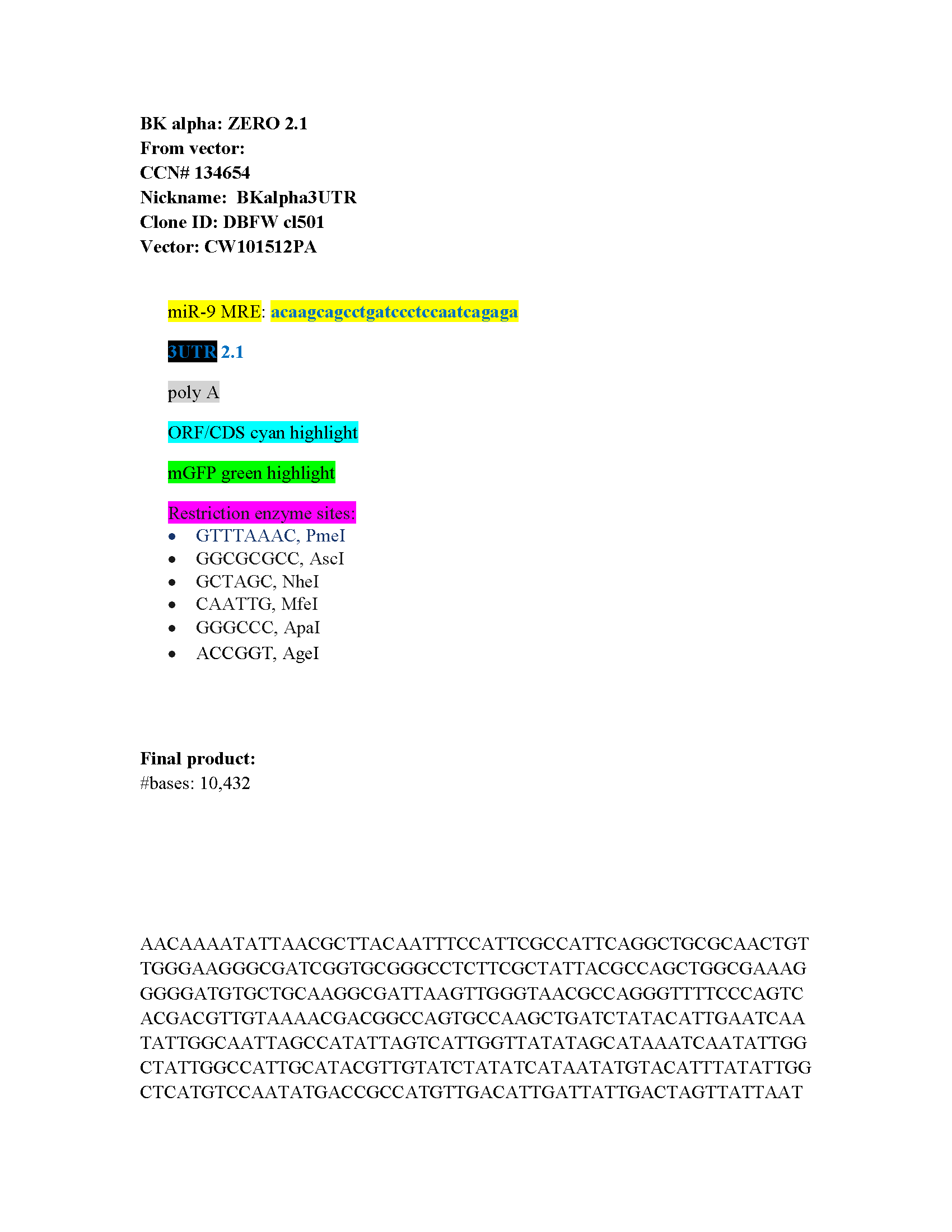

Supplement: S1 Fig — Sequence for transfected construct containing the 2.1 3’UTR variation of BK channel detailing several key portions. Yellow: miR-9 MRE, Dark Blue: 3’UTR 2.1 sequence, Grey: poly-A tail, Cian: Coding sequence for BK channel, Green: sequence of mouse GFP, Magenta: restriction enzyme sites (Blue Heron Biotech, LLC). (TIFF) [file pone.0298966.s002.tiff]

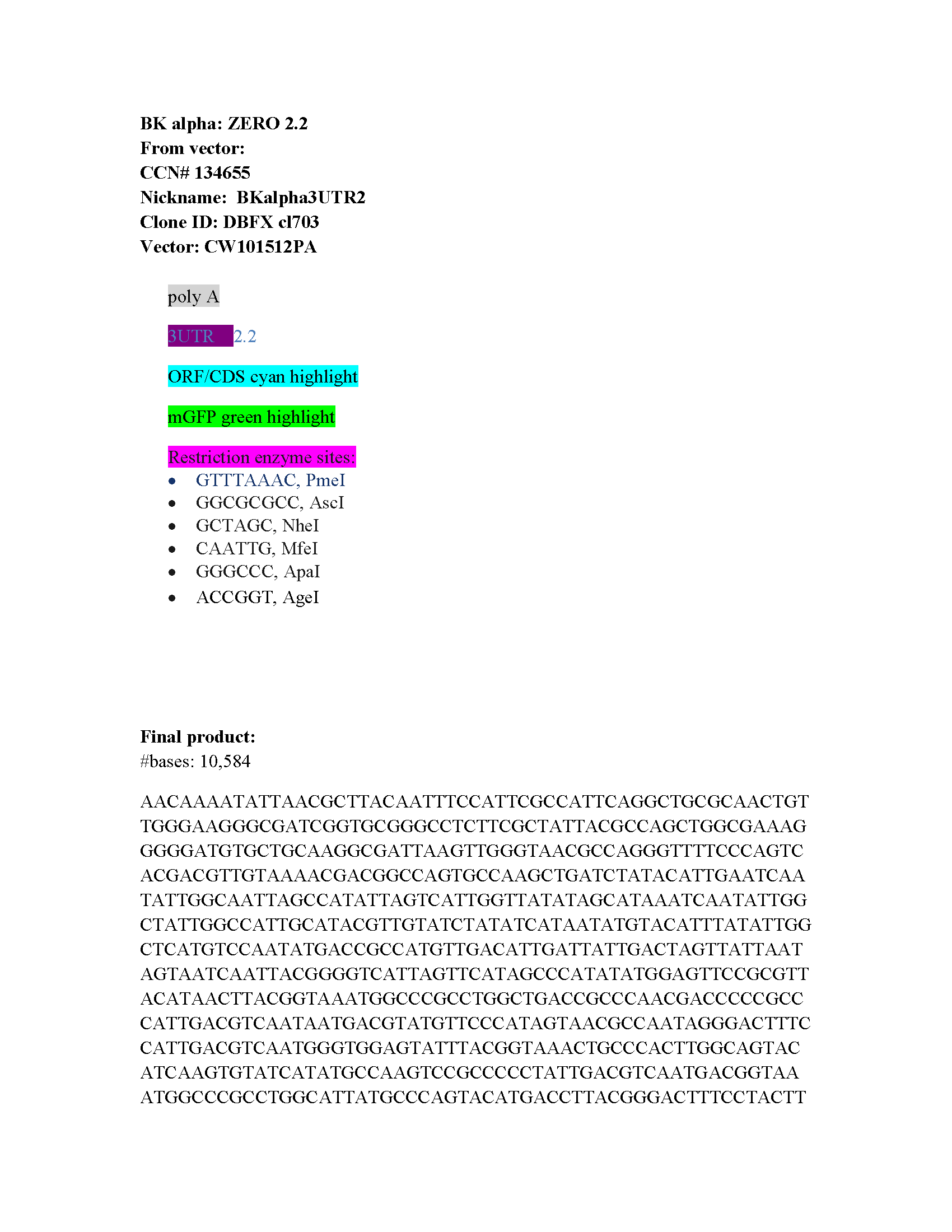

Supplement: S2 Fig — Sequence for transfected construct containing the 2.2 3’UTR variation of BK channel detailing several key portions. Dark Blue: 3’UTR 2.1 sequence, Grey: poly-A tail, Cian: Coding sequence for BK channel, Green: sequence of mouse GFP, Magenta: restriction enzyme sites (Blue Heron Biotech, LLC). (TIFF) [file pone.0298966.s003.tiff]

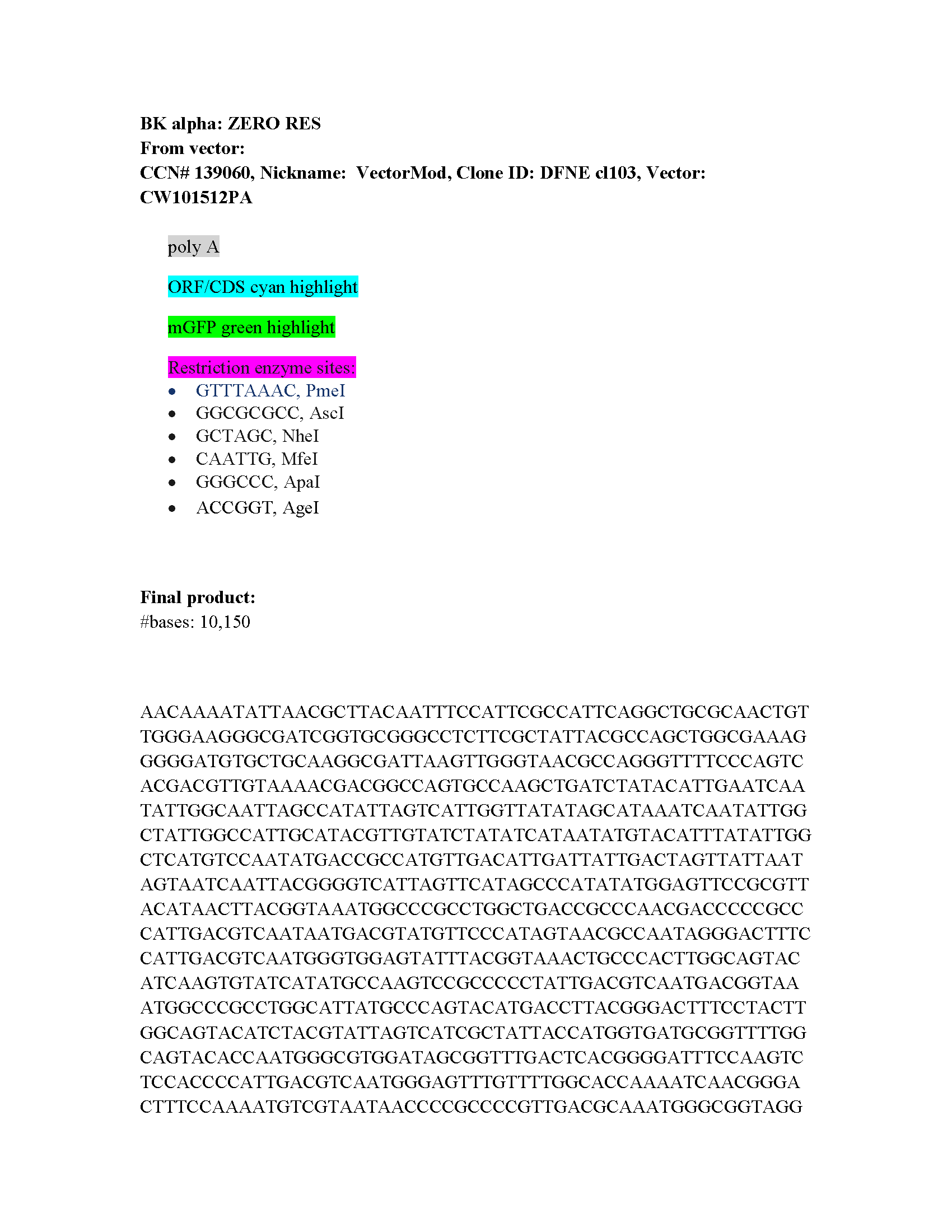

Supplement: S3 Fig — Sequence for transfected construct containing the no 3’UTR detailing several key portions. Grey: poly-A tail, Cian: Coding sequence for BK channel, Green: sequence of mouse GFP, Magenta: restriction enzyme sites (Blue Heron Biotech, LLC). (TIFF) [file pone.0298966.s004.tiff]
